# Seizures Related to Coronavirus Disease (COVID-19): Case Series and Literature Review

**DOI:** 10.7759/cureus.9378

**Published:** 2020-07-24

**Authors:** Muddasir Ashraf, Sulaiman Sajed

**Affiliations:** 1 Hospital Medicine, UnityPoint Health Trinity Rock Island, Rock Island, USA; 2 Medicine, Boston University, Boston, USA

**Keywords:** coronavirus disease, covid-19, seizures, neurological, sars-cov-2

## Abstract

Neurological manifestations are common in coronavirus disease 2019 (COVID-19) patients. We present three cases of COVID-19 patients with seizures. Two patients had a history of seizures but very well controlled. They presented with seizure activity likely triggered by COVID-19. The third patient had no history of seizures and presented with new onset of seizure activity. All these patients were routinely screened for COVID-19 on admission and tested positive on nasopharyngeal real-time reverse transcriptase-polymerase chain reaction (rRT-PCR). None of these patients had respiratory symptoms. Electroencephalography (EEG) was abnormal in all three patients. All these patients recovered and were discharged in a stable condition.

## Introduction

Coronavirus disease 2019 (COVID-19) started in Wuhan China, a city in the Hubei province, but soon became a pandemic. COVID-19 is a reality now that we have to live with, and scientists worldwide continue to learn how it affects our bodies. It was initially thought to be a respiratory disease, but we soon realized that it is not true. COVID-19 is a systemic disease that primarily affects the lungs but can affect other organs too. COVID-19 causes many neurological manifestations, including stroke, headache, altered consciousness, encephalitis, anosmia, hypogeusia, Guillain-Barre syndrome, and seizures [1-3]. These patients are usually sicker and in the intensive care unit but not in all cases [4]. Both patients with new-onset seizures and those with seizures with a previous history of seizures have been reported. Some patients may have no respiratory symptoms at all, and their initial presentation may be neurological [3]. Patients with status epilepticus have also been reported [5]. Multiple mechanisms have been proposed, and further studies are needed to be conducted to understand it better.

## Case presentation

Case 1

**Figure 1:**
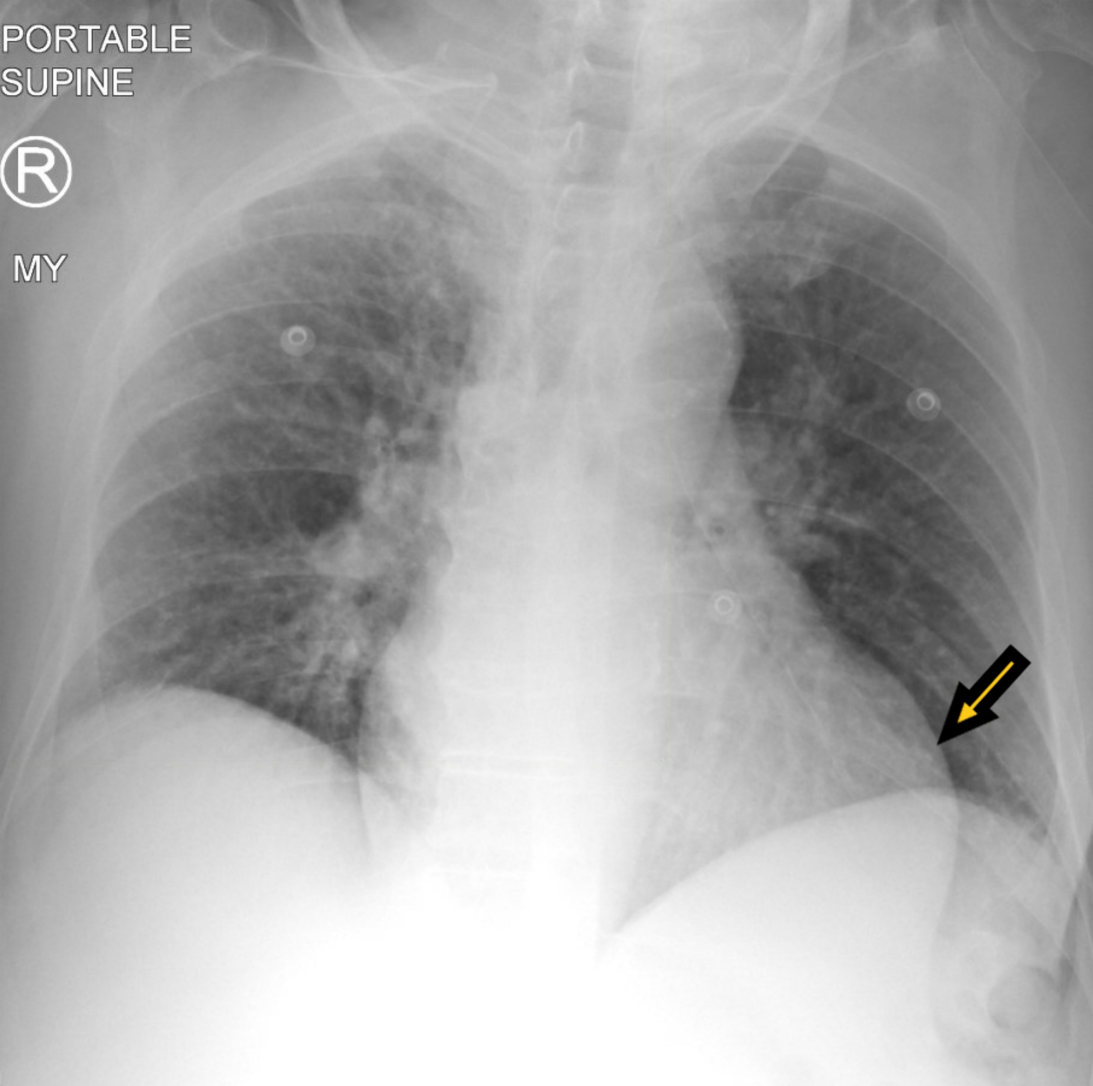
Chest X-ray showing cardiomegaly with no acute lung infiltrates.

**Figure 2:**
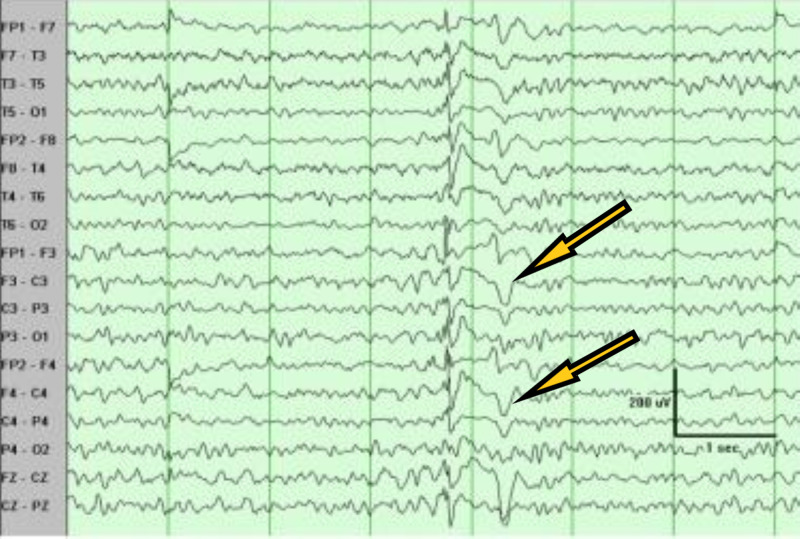
Electroencephalography (EEG) showing generalized sharps with slowing.

A 71-year-old male with a past medical history of dementia and seizures was brought by paramedics due to five episodes of witnessed generalized tonic-clonic seizures. The patient was post-ictal on arrival in the emergency and was slightly agitated later on. There was no reported history of tongue biting or loss of bladder or bowel incontinence. The patient was reportedly compliant with his antiseizure medications, which included oxcarbazepine and lacosamide. On physical examination, vital signs were stable. The patient was disoriented, agitated, and not following commands. The rest of the physical examination was unremarkable. Labs, including complete blood count and basic metabolic panel, were unremarkable except mildly elevated white blood cell count of 12.3 x 10^3^/uL. Creatinine kinase levels were normal. Real-time reverse transcriptase-polymerase chain reaction (rRT-PCR) for COVID-19 came back positive. Chest X-ray (Figure 1) showed cardiomegaly, but no acute infiltrates. Computed tomography (CT) of the head did not show any acute abnormalities. The patient was loaded with intravenous Keppra® and admitted to the hospital. Electroencephalography (EEG) (Figure 2) showed generalized sharps with slowing. The patient subsequently improved with his mentation back to the baseline. He did not have any further seizures. The patient was finally discharged with increased doses of oxcarbazepine and lacosamide.

Case 2

**Figure 3:**
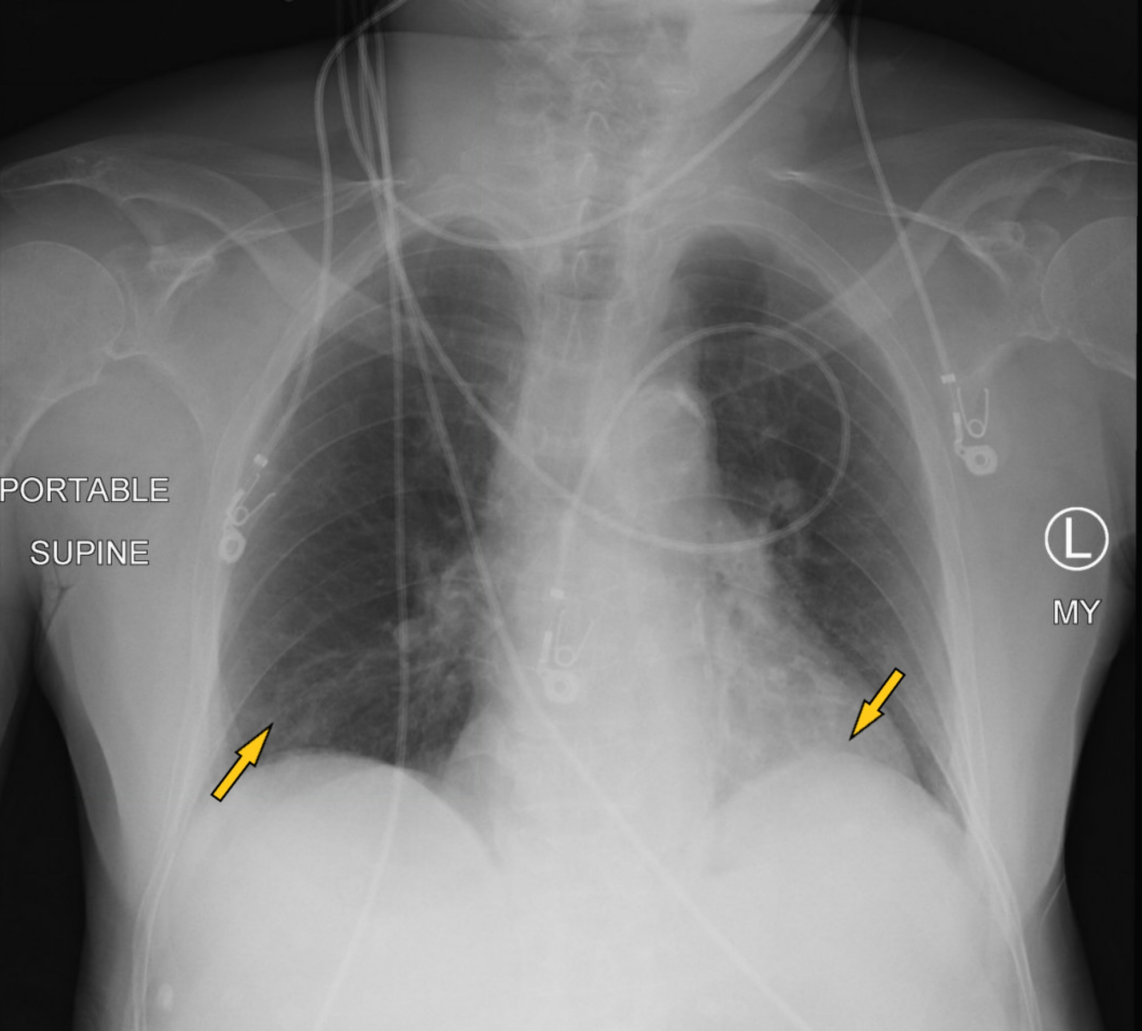
Chest X-ray showing bibasilar small airspace disease.

**Figure 4:**
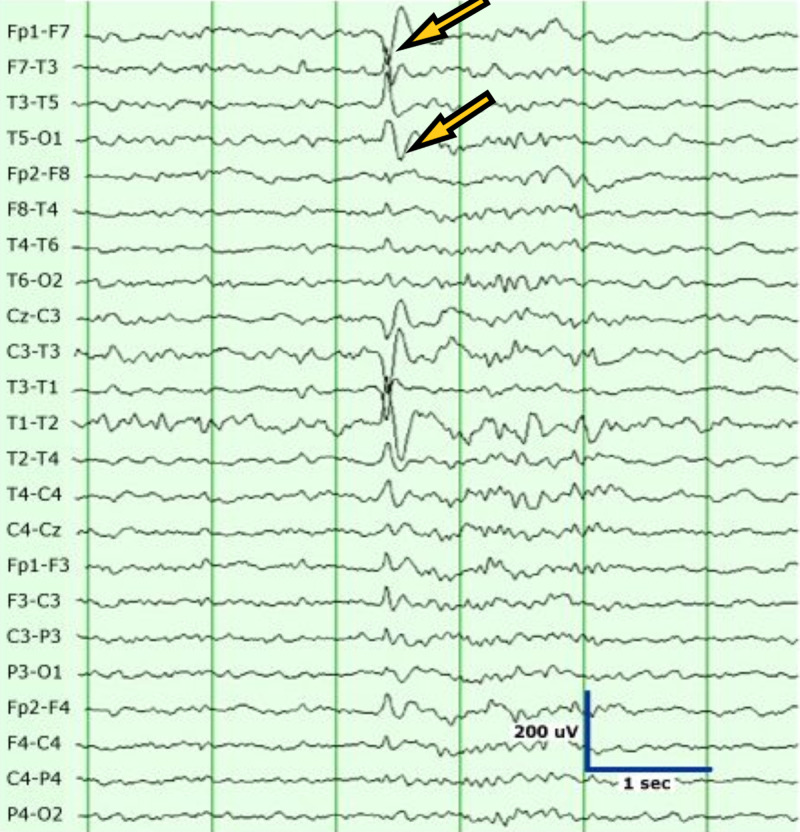
Electroencephalography (EEG) showing left-sided sharps with slowing.

A 78-year-old male resident of a nursing home with a past medical history of hypertension, diabetes type 2, and dementia presented to the hospital with the symptoms of altered mental status. The patient started having generalized tonic-clonic seizures on arrival in the emergency room. The patient did not have any witnessed seizure activity in the nursing home but had fallen with face down on the floor. The patient did receive Versed® by paramedics. The patient was unresponsive in the emergency room after the seizure. On physical examination, vitals were stable with no evidence of hypoxemia. The chest was clear to auscultation. The cardiovascular examination was unremarkable. The abdomen was soft and non-tender, and bowel sounds were positive. Neurological examination was significant for unresponsiveness, with the patient only responding to painful stimuli. Pupils were equal and reactive to light. Initial workup in the emergency room was significant for leukocytosis, metabolic acidosis with lactic acid greater than 18 mmol/L, and acute renal failure. Labs are shown in Table 1. rRT-PCR for COVID-19 was positive. Chest X-ray (Figure 3) showed bibasilar small airspace disease. CT of the head was unremarkable for any acute abnormalities. Magnetic resonance imaging (MRI) of the brain was unremarkable as well. CT of the abdomen also showed no acute abnormalities. The patient was started on intravenous fluids and empiric intravenous antibiotics. Ammonia levels were 354 umol/L on admission, and the patient was started on lactulose through a nasogastric tube for hepatic encephalopathy. Ultrasound liver did not show any evidence of liver disease. EEG (Figure 4) performed on admission did show left-sided sharps with background slowing. The patient was also loaded with intravenous Keppra on arrival in the emergency room and later on continued during the hospital course. The patient did not have any further seizures during the hospital course. The patient made a remarkable recovery with no need for ventilation and recovered back to his baseline with the resolution of acidosis and renal failure. Ammonia levels normalized as well. All other laboratory parameters also improved. The patient was finally discharged on oral Keppra back to the extended care facility.

**Table 1:** Laboratory data BUN, blood urea nitrogen; eGFR, estimated glomerular filtration rate; WBC, white blood cell count; RBC, red blood cell count; MCV, mean corpuscular volume; MCH, mean corpuscular hemoglobin; MCHC, mean corpuscular hemoglobin concentration; RDW, red blood cell distribution width; LDH, lactate dehydrogenase

Component	Value	Reference Range	Units
Sodium	149	136-145	mEq/L
Potassium	4.4	3.5-5.1	mEq/L
Chloride	107	99-109	mEq/L
CO_2_	19	19-28	mEq/L
BUN	18	7.0-18	mg/dL
Creatinine	1.6	0.7-1.3	mg/dL
eGFR	51	>60	mL/min/1.73 m²
Calcium	7.2	8.7-10.7	mg/dL
Anion gap	23	7.0-15	mmol/L
Glucose	362	70-100	mg/dL
WBC	20.7	4.8-10.8	K/uL
RBC	3.98	4.10-5.80	m/uL
Hemoglobin	10.7	12.6-17.4	g/dL
Hematocrit	33.7	37.5-50.7	%
MCV	84.8	81.0-97.0	fL
MCH	26.8	27.0-31.0	pg
MCHC	31.6	33.0-37.0	g/dL
RDW	14.8	11.5-14.5	%
Platelets	233	130-400	K/uL
Differential type	Auto		
Neutrophils	52.8		%
Lymphocytes	37.8		%
Monocytes	6.2		%
Eosinophils	2.3		%
Basophils	0.9		%
Neutrophils absolute	10.9	1.9-8.0	K/uL
Lymphocytes absolute	7.8	0.9-5.2	K/uL
Monocytes absolute	1.3	0.2-1.0	K/uL
Eosinophils absolute	0.5	0.0-0.8	K/uL
Basophils absolute	0.2	0.0-0.2	K/uL
Lactic acid, venous	>18.3	0.5-2.0	mmol/L
LDH	593	125-220	u/L
Ammonia	354	18-72	umol/L

Case 3

**Figure 5:**
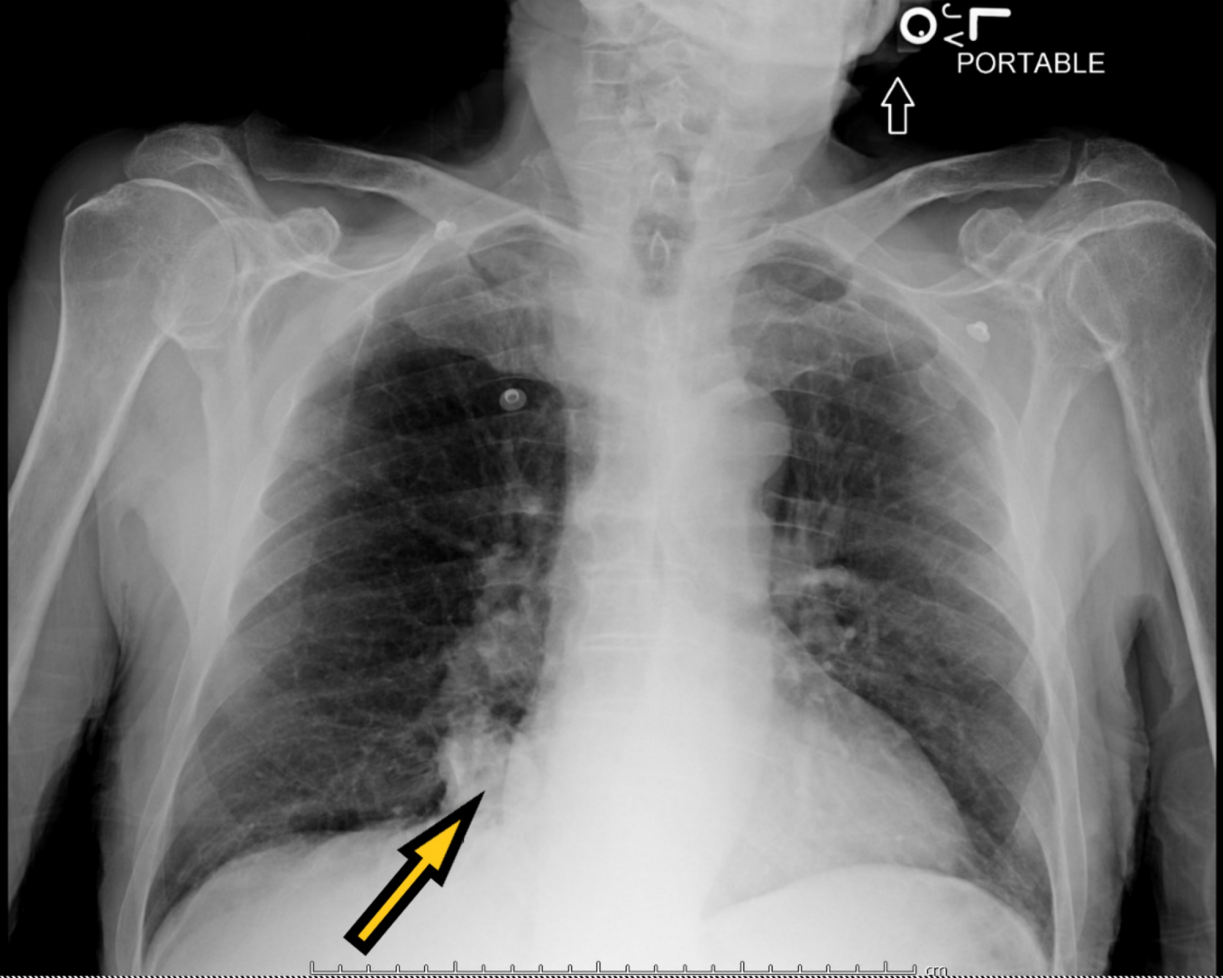
Chest X-ray showing focal consolidation in the medial right lung base.

**Figure 6:**
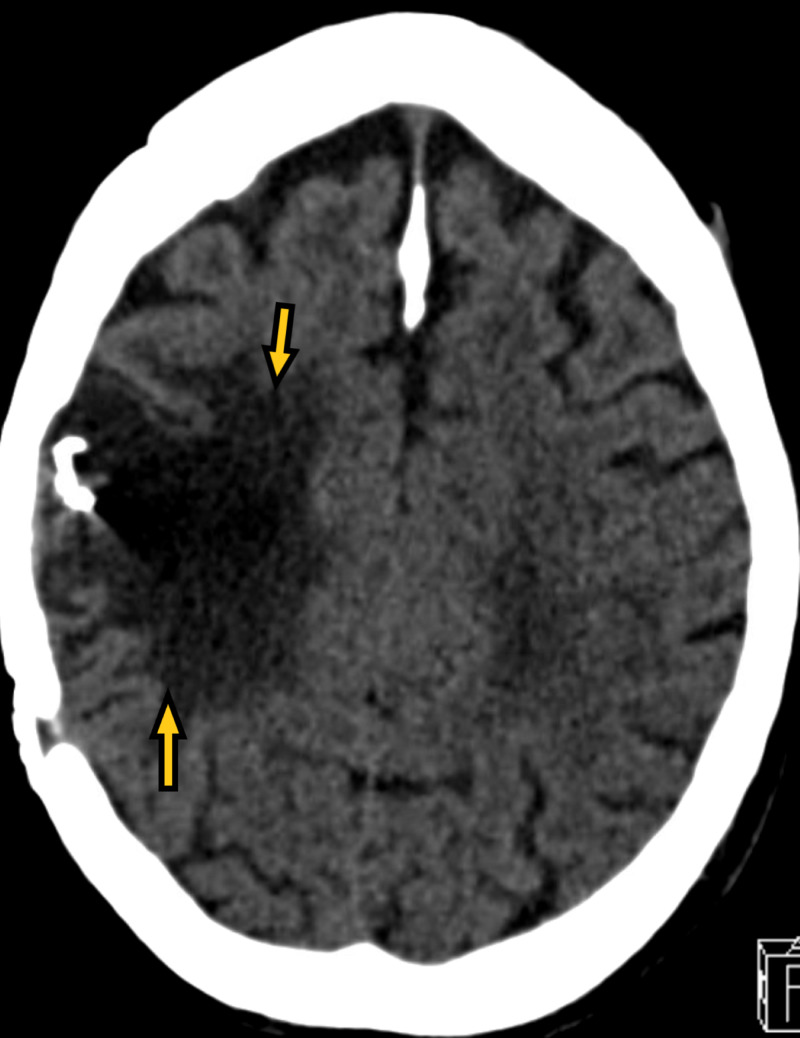
CT of the brain showing right sided encephalomalacia. CT of the brain denotes CT of the head.

**Figure 7:**
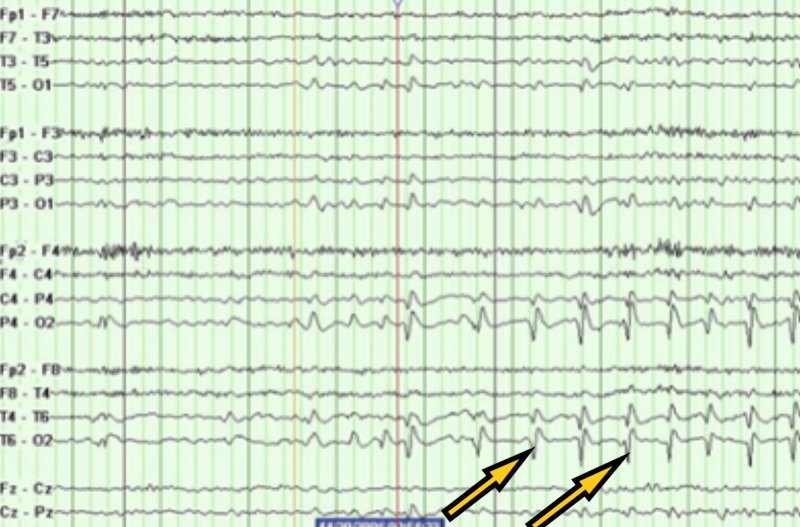
Electroencephalography (EEG) showing right-sided sharps with slowing.

A 70-year-old male resident of the nursing home with a past medical history of ischemic stroke with residual left-sided hemiparesis, seizure disorder, Crohn's disease, hyperlipidemia, and dementia on Aricept presented to the emergency room with generalized tonic-clonic seizures. The seizure was witnessed in the nursing home. The patient was post-ictal on arrival in the emergency room. The patient was also agitated afterward. The patient had no respiratory symptoms. On physical examination, vitals were stable. The neurological examination was significant for dysarthric speech and left-sided hemiparesis, and the patient was disoriented. The rest of the physical examination was unremarkable. Labs, including complete blood count and comprehensive metabolic panel, were unremarkable. Troponin, creatine phosphokinase, and C-reactive protein were within normal limits. rRT-PCR for COVID-19 came back positive. Chest X-ray (Figure 5) showed focal consolidation in the medial right lung base. CT brain (Figure 6) showed old right encephalomalacia with no acute abnormalities. EEG (Figure 7) showed slowing with right-sided sharps. The patient was loaded with intravenous Keppra in the emergency room. The patient did not have any further episodes. The patient gradually improved and was finally discharged back to the nursing home with the same regimen of lacosamide and lamotrigine, with no dose changes.

## Discussion

COVID-19 is known to cause respiratory problems such as pneumonia. However, its role in causing seizures in patients is still under study. It is reported that COVID-19 causes an inflammatory response, releasing inflammatory cytokines that play a role in acute seizures [2]. Previous studies show central nervous system (CNS) infection from the severe acute respiratory syndrome coronavirus (SARS-CoV) infection from 2003 [6]. As COVID-19 is also a part of the coronavirus family, it would share a similar pathway to that of SARS-CoV. Similar cases have arisen within patients with no history of seizures who develop a seizure [4,5]. However, a study in China with 304 patients found that seizures were not occurring within these patients, attributing any seizures seen to acute stress or hypocalcemia [7]. This study was conducted earlier in the year before COVID-19 was labeled a pandemic, which could contribute to the lower incidences of seizures. Another study contradicts the study conducted in China, showing the growing number of neurological complications associated with COVID-19 [1]. The development of headaches, encephalitis, strokes, and epileptic seizures are among the many neurological symptoms that are manifesting within COVID-19 patients [1]. Elderly patients with chronic conditions or severe patients of COVID-19 are at a higher risk of these neurological symptoms in the setting of these types of acute infections [8,9].

The genetic material of viruses can be found in nervous tissue samples [10]. While subsequent studies need to be conducted to gain evidence of COVID-19 spreading into the CNS, mechanisms have been proposed as to how the virus spreads. A proposed mechanism is that the virus can move from the olfactory nerve to the CNS [4]. This can occur within the first seven days of infection as COVID-19 quickly moves from the olfactory tract to the brain and cerebral spinal fluid [10]. This would cause the release of cytokines by the host’s immune system, leading to encephalitis. While the olfactory nerve propagation is possible within some coronaviruses, some can also penetrate the CNS through the cribriform plate of the ethmoid [1]. More studies are needed to be conducted to determine whether the propagation is done through this method. Another proposed mechanism is the hematogenous spread of the viral particles within the CNS [4]. This would be possible through the circulating lymphocytes. After infection, the circulating lymphocytes traveling to the brain would infect it, causing the inflammatory response. COVID-19 also has been seen to have a large affinity for angiotensin-converting enzyme 2 (ACE-2) [1]. The viral attachment to ACE-2 at the blood-brain barrier could be the source of viral encephalitis. These proposed mechanisms can explain how COVID-19 spreads within the CNS; however, the neurotropic pathogenic mechanism must be studied in further detail to understand the neurological effect COVID-19 has. Similar studies into the Nipah virus, which also causes neurological symptoms identical to those found in COVID-19 patients, along with research into SARS can possibly reveal information as to how COVID-19 spreads within the body [11]. As COVID-19 is now something that we have to live with, it is urgent that we must understand it more thoroughly to understand its pathogenesis.

## Conclusions

COVID-19 can cause many neurological complications including seizures. Not only can it worsen seizure control in the patients with previously well-controlled seizures, but it can also cause new-onset seizures in healthy patients. The new-onset seizures are generally present in sicker patients, as was the case in our patient. Though there are many proposed mechanisms, further research will make our understanding of its pathophysiology better.
